# Caspofungin Affects Extracellular Vesicle Production and Cargo in *Candida auris*

**DOI:** 10.3390/jof8100990

**Published:** 2022-09-21

**Authors:** Rafaela F. Amatuzzi, Daniel Zamith-Miranda, Isadora F. Munhoz da Rocha, Aline C. R. Lucena, Sharon de Toledo Martins, Rodrigo Streit, Charley C. Staats, Gabriel Trentin, Fausto Almeida, Marcio L. Rodrigues, Joshua D. Nosanchuk, Lysangela R. Alves

**Affiliations:** 1 Gene Expression Regulation Laboratory, Carlos Chagas Institute, FIOCRUZ PR, Curitiba 81350-010, Brazil; 2 Department of Microbiology and Immunology, Albert Einstein College of Medicine, Bronx, NY 10461, USA; 3 Division of Infectious Diseases, Department of Medicine, Albert Einstein College of Medicine, Bronx, NY 10461, USA; 4 Laboratory for Applied Sciences and Technology in Health, Carlos Chagas Institute, FIOCRUZ PR, Curitiba 81350-010, Brazil; 5 Programa de Pós-Graduação em Biologia Celular e Molecular, Centro de Biotecnologia, Universidade Federal do Rio Grande do Sul, Porto Alegre 91509-900, Brazil; 6 Departamento de Biologia Molecular e Biotecnologia, Universidade Federal do Rio Grande do Sul, Porto Alegre 90010-150, Brazil; 7 Department of Biochemistry and Immunology, Ribeirão Preto Medical School, University of São Paulo, Ribeirao Preto 14040-900, Brazil; 8 Microbiology Institute, Federal University of Rio de Janeiro (UFRJ), Rio de Janeiro 21941-901, Brazil

**Keywords:** *Candida auris*, extracellular vesicles, RNA, protein, drug resistance

## Abstract

Antifungal resistance has become more frequent, either due to the emergence of naturally resistant species or the development of mechanisms that lead to resistance in previously susceptible species. Among these fungal species of global threat, *Candida auris* stands out for commonly being highly resistant to antifungal drugs, and some isolates are pan-resistant. The rate of mortality linked to *C. auris* infections varies from 28% to 78%. In this study, we characterized *C. auris* extracellular vesicles (EVs) in the presence of caspofungin, an echinocandin, which is the recommended first line antifungal for the treatment of infections due to this emerging pathogen. Furthermore, we also analyzed the protein and RNA content of EVs generated by *C. auris* cultivated with or without treatment with caspofungin. We observed that caspofungin led to the increased production of EVs, and treatment also altered the type and quantity of RNA molecules and proteins enclosed in the EVs. There were distinct classes of RNAs in the EVs with ncRNAs being the most identified molecules, and tRNA-fragments (tRFs) were abundant in each of the strains studied. We also identified anti-sense RNAs, varying from 21 to 55 nt in length. The differentially abundant mRNAs detected in EVs isolated from yeast subjected to caspofungin treatment were related to translation, nucleosome core and cell wall. The differentially regulated proteins identified in the EVs produced during caspofungin treatment were consistent with the results observed with the RNAs, with the enriched terms being related to translation and cell wall. Our study adds new information on how an echinocandin can affect the EV pathway, which is associated with the yeast cell being able to evade treatment and persist in the host. The ability of *C. auris* to efficiently alter the composition of EVs may represent a mechanism for the fungus to mitigate the effects of antifungal agents.

## 1. Introduction

*Candida auris* is an emerging species characterized in 2009 when it was isolated from the ear canal of a patient in Japan [1]. This fungus has been identified rapidly in all continents except Antarctica. As with other *Candida* species, the major risk factors associated with *C. auris* disease include hospitalization, especially in intensive care settings (ICUs), central intravenous catheters, immune suppression, diabetes, renal failure and antimicrobial use, including the administration of antifungal medications [2] The fungus has a remarkable capacity to colonize patients and progress to candidemia, and it also causes additional invasive infections [3]. The mortality rate for candidemia caused by *C. auris* varies between 50 and 72% [4,5], depending on the presence of comorbidities. There are five main clades of *C. auris* identified so far with variable genetic and antifungal resistance profiles [6,7]. *C. auris* displays intrinsic resistance to fluconazole and about 30% of the isolates are not susceptible to echinocandins or polyenes [7]. At the genomic level, several virulence and resistance-related factors are present in *C. auris* that have been described in other Candida species, such as biofilm formers, transporters, phospholipases and proteases [8,9].

During infection, fungi cells secrete different types of molecules to modify the host-pathogen interaction and favor the pathogenic processes, including proteases, cytotoxic proteins and other virulence factors [10,11]. In addition to conventional pathways of cellular communication, another important more recently characterized route is the transfer of diverse microbial compounds via extracellular vesicles (EVs) [12]. By definition, EVs are cellular particles limited by a lipid bilayer and incapable of replicating, and they carry an array of functional molecules inside them [12]. EVs have been characterized in diverse species [13], with the most information being available on EVs generated by *Cryptococcus, Histoplasma* and *Candida albicans*. The payloads of fungal EVs are rich in polysaccharides, lipids, allergens, pigments, proteins, and nucleic acids, especially RNA molecules [11]. These RNA molecules have the potential for playing different roles in target cells. Overall, fungal EVs serve important roles related to virulence and disease progression, such as the production of matrix for biofilm [14] and the release of molecules associated with virulence [15], cell wall remodeling [16], and general host response and modulation [17].

In summary, *C. auris* is an emerging organism whose biology is still poorly understood, and we have proposed that the composition of EVs is linked with the fungus’ response to antifungal stress [18,19]. We and other investigators have demonstrated that *C. auris* has a dynamic response to caspofungin and other antifungal agents [20,21,22]. This study aimed to characterize alterations in the EV response of *C. auris* to caspofungin treatment.

## 2. Materials and Methods

### 2.1. Fungal Growth Conditions

The *C. auris* strains MMC1 and B8441 (Clade I) and B11244 (Clade III) were maintained at −80 °C. B8441 and B11244 were obtained from the Centers for Disease Control (CDC) (also known as CDC387 and CDC385, respectively). MMC1 is a clinical strain from a patient in The Bronx, NY [19]. The cells were thawed in Sabouraud broth and incubated at 30 °C for 24 h. Yeast cell suspensions were then plated onto Sabouraud agar plates and incubated at 30 °C for 48 h. Based on MICs, the concentrations of caspofungin used in the experiments was 12.5 ng/mL (MMC1) 10 ng/mL (B11244) and 100 ng/mL (B8441).

### 2.2. Extracellular Vesicles Isolation

One colony from each strain was inoculated into Sabouraud broth for 24 h at 37 °C at 200 rpm. Cell density was adjusted to 10^6^ cells/mL in a total volume of 400 mL with or without the addition of caspofungin for 24 h at 37 °C at 200 rpm. Afterward, the cells were centrifuged, once at 8000 rpm for 15 min at 4 °C and then at 14,000 rpm for 15 min at 4 °C. The supernatant was filtered on a 0.45 μm membrane (Milipore, Burlington, MA, USA) and concentrated to reduce the volume using an Amicon with a 100 KDa cutoff membrane. The concentrate was submitted to two rounds of centrifugation for 1 h at 150,000× *g* at 4 °C. The supernatant was discarded and the EVs were suspended in 100 µL of PBS.

### 2.3. Nanoparticle Tracking Analysis (NTA)

To determine the EVs concentration and size, we used Nanoparticle Tracking Analysis (NTA; Nanosight LM-10-Malvern Panalytical, Malvern, UK) by analyzing the light scattering pattern and Brownian motion of the particles. EV samples were diluted in PBS prior to injection. The videos were set to three runs each of 60 s, and the detection threshold was defined as 2 and camera level as 9. The data were analyzed using NTA 3.1 software (Malvern Panalytical, Malvern, UK). Total yield (EV particles/mL) was calculated based on dilution factors. A statistical analysis was performed with Minitab Statistical Software 17.0, where data were subjected to one-way variance analysis, and values were compared with a Tukey’s test with 5% probability.

### 2.4. Transmission Electron Microscopy

A total of 50 µL of EVs samples were deposited onto carbon-coated 300-mesh copper grids, incubated for 60 min at room temperature, washed with PBS, fixed with Karnovisk solution for 10 min, and then washed three times with cacodylate buffer 0.1 M. The grids were then stained with 30 µL of 5% (*v*/*v*) uranyl acetate. Excess solution was blotted off and the grids were washed with ultrapure water (18.2 Ώ) and dried overnight. Images of the EV were captured using a JEOL JEM 1400 electron microscope (JEOL Ltd., Tokyo, Japan) operated at 100 kV and magnification at 20 k or 25 k.

### 2.5. Data Analysis

The RNA was isolated and sequenced as previously described [18]. Briefly, the EV RNA was isolated with the miRNeasy kit (Qiagen, Hilden, Germany) according to the manufacturer’s instructions. To remove any contamination with DNA, we performed the DNA cleanup step (Qiagen). Afterward, the RNA was quantified by fluorimetry using a Qubit fluorometer (Thermo Fisher, Waltham USA). Finally, the libraries were constructed with 100 ng of EV RNA using the TruSeq small RNA kit (Illumina, San Diego, CA, USA), according to the manufacturer’s instructions. The sequence analysis was performed by CLC Genomics Workbench© v 20.0 (Qiagen), using the *C. auris* genome strain B8441 (GCA_002759435.2 V2) as reference. The following alignment parameters were applied: mismatch cost (2), insertion cost (3), deletion cost (3), length fraction (0.8), and similarity fraction (0.8). The sequences were trimmed to remove the internal adapter (TruSeq small RNA adapter sequence: TGGAATTCTCGGGTGCCAAGG). For the statistical analysis we used Differential Gene Expression (DGE). The transcripts expression levels were represented as Transcripts per Million (TPM) and normalized by TMM (trimmed mean of M values) [23]. For the differentially expressed transcripts, the parameters were changed threefold and with a false discovery rate (FDR) below or equal to 0.05.

For the EV mRNA identification, we combined the differential expression with read coverage by performing the map reads to reference (C_auris_B8441_version_s01-m01-r10_genomic and C_auris_B8441_version_s01-m01-r10_other_features_plus_intergenic) as follows: no masking, match score (1), mismatch cost (2), linear insertion cost (3), deletion cost (3), length fraction (0.6), similarity fraction (0.8) and global alignment. To consider the full-length mRNAs, we selected those with expression values (TPM) higher than 100 and with 5× transcript coverage.

The orthology analysis was performed by comparison with *C. albicans* genome strain SC5314 genome (assembly ASM18296v3) by Reciprocal Best Hit (RBH). The genes that presented more than 30% of identity were considered for the analysis. For the functional studies, DAVID 6.8 software [24] was used for the Gene Ontology terms and the String for protein interaction network and enrichment analysis [25]. For enrichment analysis, a Fisher’s exact test was applied and considered only the terms with *p*-value ≤ 0.05.

The genomic sequence and transcript annotation was obtained from the NCBI’s Cand_auris_B11221_V1 assembly. Initially, the small RNA-seq read libraries were quality filtered using fastx-toolkit’s fastq_quality_filter (v0.0.14) (A. Gordon, G.J. Hannon, Fastx-toolkit. FASTQ/A short-reads pre-processing tools. (Unpublished) http://hannonlab.cshl.edu/fastx_toolkit 5 (2010)), removing reads with any nucleotide with a quality score lesser than 20 (-q 20 -p 100). In order to confirm library strandness orientation, we first aligned the libraries against the *C. auris* genome sequence using HISAT2 (v2.2.1) [26] with—very-sensitive alignment and converted the alignment files to bam format using samtools (v1.13) [27]. Then we converted the transcript annotation GFF file to BED format using BEDOPS’ gff2bed (v2.4.39) [28] and extracted the coordinates of rRNA and tRNA genes, and provided this rRNA and tRNA BED in combination with the bam alignment file to RSeQC’s infer_experiment.py (v3.0.1) [29], confirming that the libraries were forward-stranded. In order to count the number of reads aligning to each gene’s forward and reverse strand, we generated a “reverse transcript” GFF file from the transcript annotation GFF to specifically count genes aligned to each strand. Next, we aligned the libraries against the genomic sequence again using hisat2 using --very-sensitive and --rna-strandness F, converted the alignment files to bam and sorted them using samtools, and counted the reads aligning to each strand (each of the different transcript strand GFF files) of each gene using HTseq’s htseq-count (v0.13.5) [30] with the options -r pos -s yes -t gene -m intersection-nonempty and using both --nonunique none and all.

In order to obtain the sequence of each antisense sRNA, we first recovered the gene sequence from the genome using BEDtools’ getfasta (v2.30.0) [31] and aligned our libraries against these sequences using hisat2. We then filtered the alignment files using samtools, extracting alignment files from reads aligning to the sense strand as well as the antisense strand. The generated files were sorted with samtools, converted back to fastq using BEDtools’ bamtofastq, the fastq files were converted to fasta format using seqtk seq (v1.3) (https://github.com/lh3/seqtk), parsed using Biopython’s SeqIO (v1.78) [32], and at last the sequences of each sRNA was recovered from the fasta file and redundancy was removed. We then compared the sequences recovered from sense and antisense strands and removed common sequences, generating a list of antisense-only sRNA sequences, which was used to create a fasta file. To identify possible targets for the identified antisense sRNAs, we aligned the sRNAs against the transcripts from *C. auris* (for sRNAs identified from intracellular sRNA libraries) assembly transcripts, for sRNAs identified from EVs sRNA libraries) using BLASTn (2.6.0) [33] with -strand minus option in order to align in an antisense manner to evaluate base complementarity.

The RNA-seq data is available at the Sequence Read Archive (SRA) database under the accession number (SRA: SRP295539 BioProject: PRJNA682185).

### 2.6. Proteomic Analysis

For the proteomic analysis, the EVs were isolated in three independent replicates of each condition. Isolated EV samples were dried in Speed Vac equipment and then a lysis buffer (100 mM 1 M DTT, 100 mM 1 M Tris HCl at pH 7.5, 4% SDS and 1% Triton X-100) was added to them. The samples were incubated for 10 min at room temperature. The protein extracts were centrifuged for 5 min at ~14,000× *g* and the supernatants were transferred to new tubes.

Protein extracts were added to a 15% polyacrylamide gel with sample buffer (40 mM 1 M Tris-HCl at pH 6.8, 1% SDS, 14.7 M 2.5% betamercaptoethanol, 6% glycerol, 0.005% bromophenol blue). Prior to the run, each sample/sample buffer mix was incubated at 95 °C for 3 min. The gel was stained with silver nitrate. To obtain the peptides, each lane was cut in small pieces and was destained with silver decoloration solution (100 mN sodium thiosulfate and 30 mM ferrocyanide). The gel pieces were washed with 0.05 M ammonium bicarbonate (ABC) until they were completely transparent. Sequentially, the samples were dehydrated with ethanol, reduced with 10 mM DTT, alkylated with 50 mM iodacetamide and proteins were digested with 12.5 ng/µL trypsin solution in ABC. Peptides were extracted with extraction solution (30% acetonitrile-ACN, 3% trifluoracetic acid) and ACN 100%. The peptides were desalted with C18 Stage Tips. 

The samples were fractioned through liquid phase liquid chromatography, with 130 mg Sep Pack C18 columns. Elutions were performed with different concentrations of ACN and the fractions were dried with Speed Vac. Fractions were analyzed by ESI MS/MS. The experiments were performed with and EASY-nLC 1000 (Thermo Scientific, Waltham, MA, USA) chromatographer coupled with the mass spectrometer LTQ Orbitrap XL ETD (Thermo Scientific) equipped with a 2.3 kV ionization source at 250 nl/min.

The mass spectrometry data were analyzed using MaxQuant software (v1.5.5.1). Peptide identification was carried out through research among the reference *C. auris* sequence (UP000230249) at the Uniprot database. The research parameter included variable modifications at the *N*-terminal acetylation, methionine oxidation and carbamidomethylation of the cysteine residues. For validation, peptides were required to have at least 7 amino acids. The generated data was analyzed with Perseus software (v.1.5.0.31).

## 3. Results

### 3.1. Characterization of EVs Produced by C. auris Treated with Caspofungin

**Figure 1:**
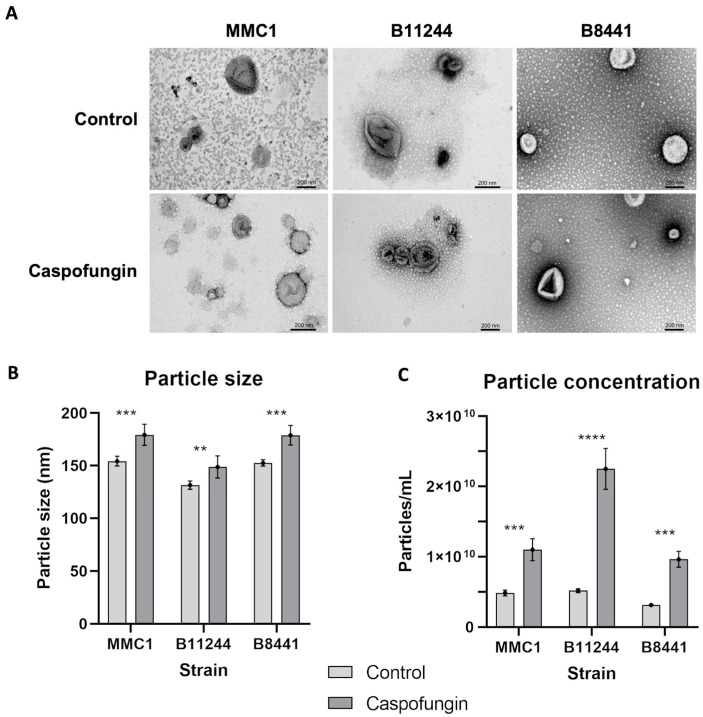
Characterization of *Candida auris* extracellular vesicles (EVs). (**A**) Transmission electron microscopy images of EVs derived from MMC1, B11244 and B8441 strains under control conditions and caspofungin treatment. bar = 200 nm. (**B**) Particle size distribution bar chart for MMC1, B11244 and B8441 EVs from control (light grey) and caspofungin treatment (dark grey). The average EV size (*y*-axis) is indicated in nm. The *x*-axis indicates the strains studied, where *** *p* < 0.005 and ** *p* < 0.05, by individual t-tests followed by Bonferroni’s multicomparisons test for three independent experiments. (**C**) A particle concentration bar chart related to EVs concentration calculated the three strains from control and caspofungin treatment; the *y*-axis indicates the particles concentration, *** *p* < 0.001 and **** *p* < 0.0001, by one-way Anova followed by Bonferroni’s multicomparisons test for three independent experiments. The EV size is indicated in nm.

In order to evaluate the response elicited by caspofungin related to EV production and composition, we used three distinct strains belonging to the clades I (B8441 and MMC1) and III (B11244). Under both control and antifungal-treatment conditions, *C. auris* EVs displayed the common morphology of “cup-shaped” lipid bilayered vesicles of typical sizes (Figure 1) [19]. In addition, nano-tracking particle analysis showed that the EVs isolated from the three strains displayed similar sizes and morphologies from cultures treated or not with caspofungin, indicating that the detection of EV-like particles in the drug-treated cultures was not a consequence of the leakage of intracellular organelles (Figure 1). However, the amount of EVs isolated doubled from cultures subjected to caspofungin treatment (Figure 1). In order to investigate if this increased amount of EVs observed upon caspofungin treatment was specific to caspofungin or a general antifungal response, we treated the B11244 strain with amphotericin B under subinhibitory conditions. However, no alteration in the amount of EVs was observed during amphotericin B (Supplemental Figure S1), indicating that the increased EV production or release was specific to the echinocandin. In addition, we have previously shown that the cell viability is not affected under these growth conditions, reinforcing the increased amount of EVs as a specific response [20].

### 3.2. Caspofungin Treatment Led to Alteration in the EV RNA Content

Caspofungin treatment resulted in a change in the composition of RNA in EVs. Distinct classes of RNA molecules were identified as full-length mRNAs and most of the RNAs identified are ncRNAs smaller than 200 nucleotides that included anti-sense RNAs, tRNAs, other ncRNAs and rRNA, as described in other fungi species [34].

**Figure 2:**
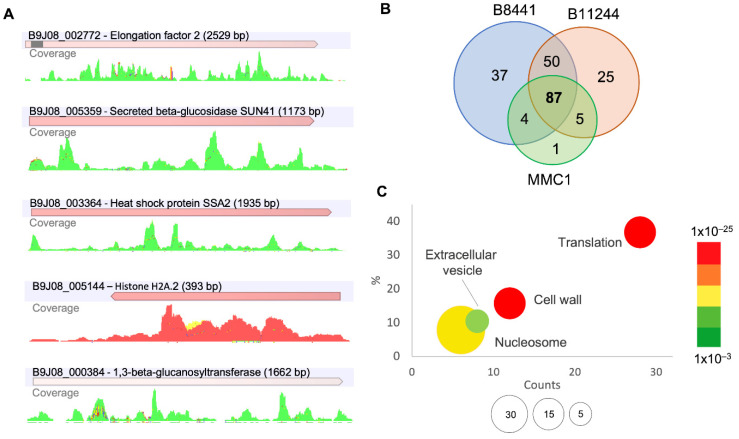
mRNAs present in *C. auris* EVs. (**A**) Reads coverage profile of transcripts enriched in EVs isolated after caspofungin treatment; forward reads (green), reverse reads (red) and non-specific match (yellow). (**B**) Venn chart of the mRNAs present in the EVs from the strains used in this study. (**C**) Gene ontology bubble chart, with the common transcripts identified in the EVs from the three strains, with the terms enriched in the presence of caspofungin. *X*-axis, the number of counts for the terms identified, *Y*-axis, the percentage of terms in the analysis; the bubble size reflects the fold-enrichment of the term after Fisher-exact test was applied and the color code reflect the *p*-value of the terms.

In order to identify full-length mRNAs, we filtered transcripts with a 5× of minimum reads coverage (Figure 2A). In total, 87 transcripts were identified as common to all the strains, 59 were detected in at least two strains, 37 were abundant in the B8441 strain, 25 mRNAs were found in the B11244 and one was found in the MMC1 strain (Figure 2B and Tables S1–S5). For the common transcripts, cell wall, nucleosome core, and ribosome, the enriched terms, and the identification of ribosomal proteins, is similar to what was observed for the transcriptome when whole yeast cells were treated with caspofungin [20] (Figure 2C). For the transcripts common to at least two strains, we observed translation related transcripts, heat shock and chaperones (Table 1). For the specific transcripts for the B8441 strain, the enriched terms were posttranslational modification, protein turnover, chaperones, and nucleus; for the B11244 and MMC1, no specific enrichment was observed. The RNA-seq data was previously validated by real-time quantitative PCR (qPCR) on B8441, B11244 and MMC1 strains under control conditions and with caspofungin treatment [18]. In addition, we compared the whole cell transcriptome treated with caspofungin with the transcripts identified in the EVs. By selecting the most expressed transcripts in the cell upregulated upon caspofungin treatment, we observed that 30% of them were also identified in the EVs. The majority of the mRNAs that were more abundant in the EVs were not related to highly expressed transcripts, indicating the active mechanism of EV cargo selection.

**Figure 3:**
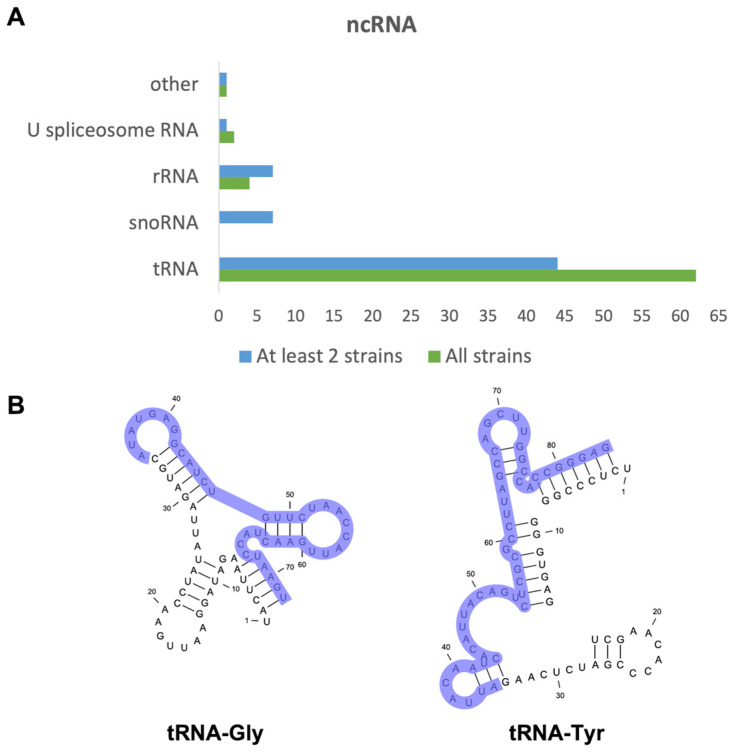
ncRNAs present in *C. auris* EVs. (**A**) Bar chart describing the most prevalent classes of ncRNAs identified in the EVs in the B8441, B11244 and MMC1 strains in green and the ncRNA identified in at least two strains in blue. (**B**) tRNA structure and sequence for the tRNA-Gly and tRNA-Tyr. Light purple highlights the region sequenced in our analysis, demonstrating that they are tRNA-derived fragments (tfRNAs).

The ncRNAs represented the bulk of RNA molecules in the EVs. When comparing the three strains, 78 identified ncRNAs were common among them. A total of 80 ncRNAs were identified in at least two strains (Figure 3A). For the B8441 strain, 43 ncRNAs were exclusively identified in this strain, for B11244 only five ncRNAs were detected and 10 were identified for the MMC1 strain. For the B8441 and MMC1 strains, tRNAs were the most abundant ncRNA identified. Most of them were tRNA-derived fragments (tfRNAs), as already characterized in other organisms (Figure 3B and Tables S1–S5) [12].

**Figure 4:**
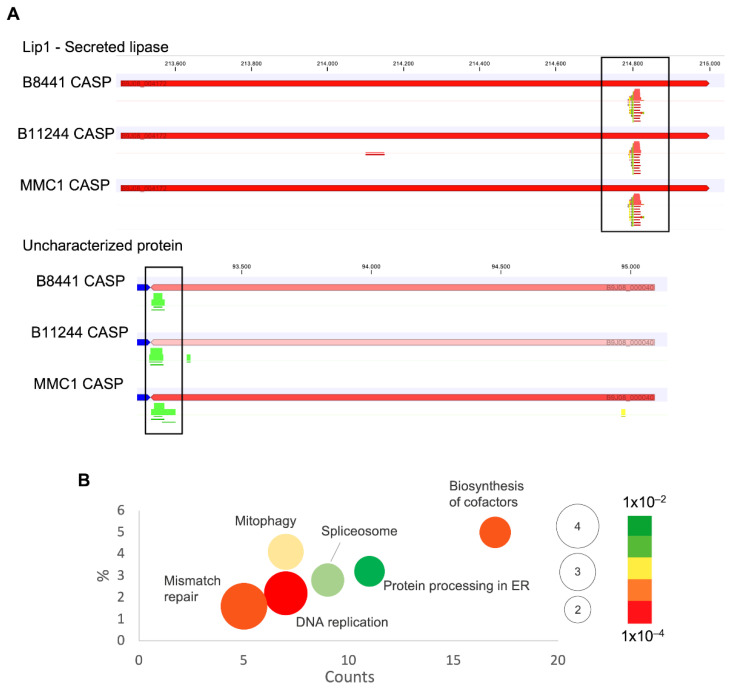
anti-sense RNAs present in *C. auris* EVs. (**A**) Specific regions of reads aligning in transcripts from EVs isolated after caspofungin treatment, in red reverse reads, and in green forward reads. (**B**) Gene ontology bubble chart of the targets of the asRNAs identified in the EVs from at least two of the studied strains, with the terms enriched in the presence of caspofungin. *X*-axis, the number of counts for the terms identified; *Y*-axis is the percentage of terms in the analysis, and the bubble size reflects the fold-enrichment of the term after a Fisher’s-exact test was applied and the color code reflects the *p*-value of the terms.

In addition, we also detected in our RNA-seq mRNA fragments that presented low coverage, with reads aligning in specific regions and, interestingly, in the complementary strand of the mRNA (anti-sense RNA-asRNA) (Figure 4A). The length of the fragments varied from 15 to 55 nt, with 11% of the sequences of 51 nt in length. These asRNAs are produced from exons and they act by regulating the expression of the correlated mRNA. This result is in agreement with our observation of short anti-sense reads in the exonic regions of the transcripts. It is possible to hypothesize that these RNA fragments carried by the EVs can regulate expression of their own mRNAs in response to a specific condition, which, in this study, was caspofungin treatment. The numbers of anti-sense fragments during the antifungal treatment were 10 times more abundant than the control for all the strains studied (Table S6). We also observed an enrichment for distinct pathways of the asRNAs; when comparing the three strains upon caspofungin treatment, the pathways for DNA replication, mismatch repair and spliceosome were enriched, and mitophagy—yeast, glycolysis/gluconeogenesis and protein processing in endoplasmic reticulum were common to at least two strains (Figure 4B).

### 3.3. EV Protein Composition Was Altered upon Caspofungin Treatment

In addition to RNA composition, we further investigated if caspofungin treatment would change the composition of EV proteins. We observed a difference in the EV protein abundance amongst strains and also in response to caspofungin.

**Figure 5:**
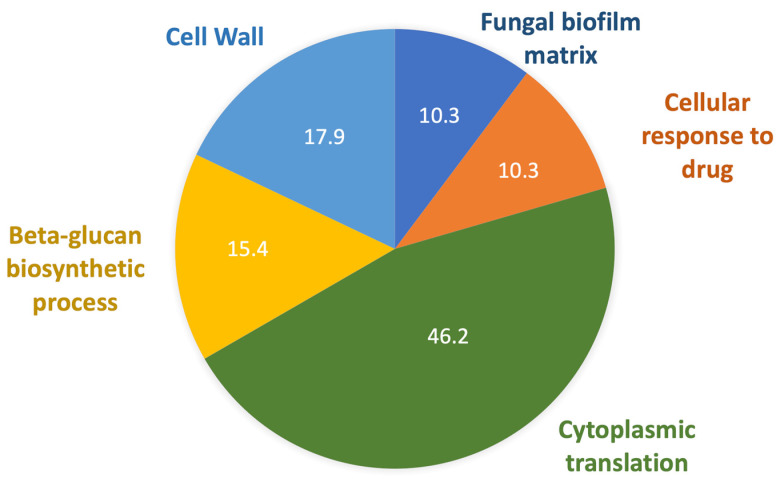
Proteins present in *C. auris* EVs. Pie chart of gene ontology terms identified in the EVs from all the strains enriched when the cells were treated with caspofungin.

After data analysis with the *C. auris* reference Uniprot sequence (UP000230249), considering proteins identified in at least two replicates, 505 proteins were identified. To obtain the proteins differentially expressed in the caspofungin treatment, control data from the different strains were pooled, and these results were used for comparison. Fold Change of 2 and *p*-value 0.05 was used as cutoff. Under these terms, 67 proteins were considered differentially expressed (Table S7), 34 proteins in B8441, 23 proteins in B11244, 34 proteins in MMC1. Among these proteins, five were identified in common between the B8441, B11244 and MMC1 strains, one protein was common between the B11244 and B8441 strains, 16 proteins were common in B8441 and MMC1, one protein was common in B11244 and MMC1, 16 proteins were exclusive in strain B8441, B11244 and MMC1 s (Table 2). The gene ontology and metabolic pathways enriched in caspofungin derived EVs were ribosome/translation, cell wall, response to drugs, biofilm formation and Beta-glucan biosynthetic process (Figure 5). In addition, we compared the cell vs. EVs protein composition and abundance [20]. Similar to the RNA-seq, approximately 21% of the EV proteins were detected in the cell after caspofungin treatment, again indicating that some sort of selection is occurring and directing the proteins to the vesicles (Table 2).

## 4. Discussion

Caspofungin treatment increased EVs release in *C. auris*, and a similar result was described in *Saccharomyces cerevisiae*, in which caspofungin treatment increased EV production [16], which is congruent with our observation.

Next, we sought to investigate the response to caspofungin related to the RNA content of *C. auris* EVs. Compared to control conditions, many transcripts were enriched in caspofungin EV-treated, as QDR3, a drug transport regulator, and it might suggest a communication in antifungal response. In *C. albicans*, QDR3 is associated to biofilm formation and virulence [35]. Another transcript was MP65, which encodes a β-glucanase mannoprotein, one of the major components of the *C*. *albicans* biofilm matrix [36]. It is also important to maintain cell wall integrity and adherence to the host-cell, representing a relevant virulence factor in *C. albicans* [37]. Heat shock proteins (HSP) are important in responding to a variety of cellular stresses. In addition, studies have demonstrated that HSP can be associated to drug resistance in *C. albicans* [38]. The presence of HSP transcripts can act by communicating stress signals to other cells, to overcome stress and promote cell survival. Sun41 is a cell surface beta-glucosidase involved in cell cycle, cell wall biogenesis, host cell adhesion, and biofilm formation, which has an important role in the host-pathogen interaction [39]. This result suggests that EV-mRNAs play a role in drug-induced stress signaling and cell-wall maintenance. The pH-responsive protein 2 (PHR2), also known as 1,3-beta-glucanosyltransferase, is an important component of the cell wall architecture. In *C. albicans*, PHR2 had an increased expression level after caspofungin treatment, and this protein was also detected in high levels in caspofungin-resistant isolates [40]. Our result reinforces the relevant role of PHR2 in responding to caspofungin in distinct *Candida* species. In addition, when we compared the cellular RNA with the transcripts identified in the EVs. For *C. auris*, as shown in other studies in distinct organisms and cell types, the most abundant cellular transcripts were not necessarily identified in the EVs, suggesting an active mechanism of EV cargo selection [41,42,43,44,45].

The studies investigating the role of ncRNAs in eukaryotic pathogens are scarce. In the parasites *Plasmodium falciparum* [46] and *Schistosoma* [47], virulence-related ncRNAs present differential expression patterns depending on the sexual stage of the organism and are related to the immune escape and survival during infection [46]. In fungi, ncRNAs are also associated with infection and virulence [48]. We showed that the tRNA-halves were the prevalent ncRNA molecules identified in the EVs, and these fragments have already been described in EVs derived from distinct organisms [49,50,51]. This pattern was also observed in our previous studies of *C. albicans*, *C. neoformans* and *S. cerevisiae* [36].

Antisense transcription is a conserved and important regulatory pathway, occurring in archaea, prokaryotes, and eukaryotes. Anti-sense RNA (asRNA) can act in all the steps of the RNA cycle, including transcription, translation, and RNA turnover [52]. The asRNA are versatile molecules; they can be short (<200 nt) or long (>200 nt) and also classified as both cis- and trans- elements, with an activating or repressing role, depending on the targets. One of the functions described for the asRNAs is in chromatin remodeling, by pairing with the sense region leading to a modification of the chromatin structure [53,54]. The important role of asRNAs in RNA metabolism in distinct fungal species, and their presence in fungal EVs are beginning to be described. However, their role in cell communication needs to be explored further.

We further studied the protein content of *C. auris* EVs upon caspofungin treatment and related these findings to that of the RNA analysis. Most peptides identified were present in the three isolates studies and associated with ribosome pathways and biogenesis and stress response, with many HSPs. Although it is indeed interesting to find a high number of ribosome-related proteins enriched after caspofungin treatment, these results are not isolated, as other studies have found an increased ribosomal protein content after yeast cells were treated with antifungals [18,19,20]. The ribosomal 40S proteins have previously been identified in *Histoplasma capsulatum* EVs [45] and in *Candida glabrata, Candida tropicalis* and *Candida parapsilosis* EVs [55]. HSP 82 is a chaperone involved in protein folding, and it has already been identified as a virulence and pathogenicity-related homolog in *Paracoccidioides brasiliensis* and *C. albicans* [56,57]. Many of the proteins identified in *C. auris* EVs are described as immunogenic in other *Candida* species, and their presence in the EVs might induce an altered immune response in the host cell [58,59,60,61]. The proteins described as immunogenic are fructose-bisphosphate aldolase, guanine nucleotide-binding protein subunit beta-like protein, ATP synthase subunit alpha, ATP-dependent RNA helicase eIF4A, alcohol dehydrogenase 1, isocitrate dehydrogenase [NAD] subunit-mitochondrial, and glyceraldehyde-3-phosphate dehydrogenase. In addition to the immunogenic profile, the ATP-dependent RNA helicase eIF4A (TIF1), ATP synthase subunit alpha (ATP1) and fructose-bisphosphate aldolase (FBA1) have been implicated in stress and antifungal response, and ATP1 and FBA1 were previously shown to be highly expressed in response to ketoconazole and flucytosine [62,63]. The proteomic results suggest that the EV proteins can play a role in antifungal and stress responses.

In summary, these results provide information on how an echinocandin can affect the EV pathway, which is associated with the yeast cell being able to evade the treatment and persist in the host. The ability of *C. auris* to efficiently alter the composition of EVs may represent a mechanism for the fungus to mitigate the effects of antifungal agents. This information expands our understanding of fungal EV biology and drug resistance, a serious and emerging global threat.

## Figures and Tables

**Table 1:** Gene ontology terms of EV-mRNAs enriched upon caspofungin treatment in at least two strains (top) or exclusive to B8441 strain (bottom).

At Least 2 Strains					
Term	Count	%	*p*-Value	Fold Enrichment	Fisher Exact
cytoplasmic translation	4	14.8	2.1 × 10^–4^	31.3	5.9 × 10^–6^
regulation of translational fidelity	2	7.4	2.9 × 10^–2^	65.3	3.7 × 10^–4^
cellular response to unfolded protein	2	7.4	3.8 × 10^–2^	48.9	6.8 × 10^–4^
translational elongation	2	7.4	4.8 × 10^–2^	39.2	1.1 × 10^–3^
chaperone mediated protein folding requiring cofactor	2	7.4	6.6 × 10^–2^	28	2.2 × 10^–3^
Protein processing in endoplasmic reticulum	4	14.8	2.6 × 10^–2^	5.7	4.3 × 10^–3^
Ribosome	4	14.8	7.4 × 10^–2^	3.8	1.8 × 10^–2^
**B8441**					
Term	Count	%	*p*-Value	Fold Enrichment	Fisher Exact
Posttranslational modification, protein turnover, chaperones	2	16.7	9.5 × 10^–2^	10.5	9.0 × 10^–3^
nucleus	4	33.3	7.5 × 10^–2^	3.2	2.3 × 10^–2^

**Table 2:** Differentially expressed proteins identified in *C. auris* EVs after caspofungin treatment comparing different strains.

Strains	Interpro ID	Product	Name
B11244 B8441 MMC1	A0A2H0ZD48	Fructose-bisphosphate aldolase	FBA1
	A0A2H0ZQE9	Mitochondrial phosphate carrier protein	MIR1
	A0A2H0ZVW8	Phosphotransferase	HXK2
	A0A2H0ZCU8	Mitochondrial outer membrane protein porin	POR1
	A0A2H0ZV45	14-3-3 protein homolog	BMH1
B11244 MMC1	A0A2H0ZM99	Heat shock protein SSA2	SSA2
B8441 MMC1	A0A2H0ZFC0	Guanine nucleotide-binding protein subunit beta-like protein	ASC1
	A0A2H1A2B0	ATP synthase subunit alpha	ATP1
	A0A2H0ZES9	Transaldolase	TAL1
	A0A2H1A3A4	SEC14 cytosolic factor	SEC14
	A0A2H0ZQK7	Translation elongation factor EF1B gamma	CAM1
	A0A2H0ZIR0	Adenosylhomocysteinase	SAH1
	A0A2H0ZSF0	40S ribosomal protein S16	RPS16A
	A0A2H1A6I3	ATP-dependent RNA helicase eIF4A	TIF1
	A0A2H0ZVC9	60S ribosomal protein L9-B	RPL9B
	A0A2H0ZQS3	D-fructose-6-phosphate amidotransferase	GFA1
	A0A2H0ZCD6	40S ribosomal protein S2	RPS21
	A0A2H0ZDC5	40S ribosomal protein S0	RPS0
B11244 B8441	A0A2H1A5H4	Peroxiredoxin TSA1	TSA1
MMC1	A0A2H0ZKB9	Ribosomal 60S subunit protein L14B	RPL14
	A0A2H0ZRL1	40S ribosomal protein S11-A	RPS11A
	A0A2H0ZKV5	Elongation factor 3	CEF3
	A0A2H0ZS48	40S ribosomal protein S24	RPS24
	A0A2H1A3M0	60S ribosomal protein L18-A	RPL18
	A0A2H0ZD81	60S ribosomal protein L6	RPL6
	A0A2H0ZFX1	60S ribosomal protein L7	RPL7
	A0A2H0ZW29	40S ribosomal protein S3	RPS3
	A0A2H1A5Q8	60S acidic ribosomal protein P0	RPP0
	A0A2H0ZYM3	Ribosomal protein L15	RPL15A
	A0A2H0ZYS2	40S ribosomal protein S13	RPS13
	A0A2H1A6Z0	GTP-binding nuclear protein	GSP1
	A0A2H0ZMQ5	Heat shock protein SSA2	CAR2
	A0A2H0ZN06	Ornithine aminotransferase	TKL1
	A0A2H1A937	ADP, ATP carrier protein	T9
	A0A2H0ZM29	40S ribosomal protein S8	RPS8A
B11244	A0A2H1A4W2	1,3-beta-D-glucan-UDP glucosyltransferase	GSC1
	A0A2H0ZP18	Multidrug resistance protein 1	MDR1
	A0A2H1A5R8	MFS domain-containing protein	HGT10
	A0A2H0ZW02	Peptidase A1 domain-containing protein	
	A0A2H0ZNS4	GTP-binding protein RHO1	RHO1
	A0A2H1A160	Ras-related protein SEC4	SEC4
	A0A2H0ZKL2	AMP-binding domain-containing protein	FAA4
	A0A2H0ZDG8	Uncharacterized protein	
	A0A2H0ZKV9	Plasma membrane ATPase	PMA1
	A0A2H0ZFX3	1,3-beta-glucanosyltransferase	PGA4
	A0A2H1A5H8	Ubiquitin-40S ribosomal protein S27a	UBI3
	A0A2H0ZVC5	Protein transport protein SSO2	SSO2
	A0A2H0ZXY4	Beta-glucan synthesis-associated protein	KRE6
	A0A2H1A7S4	1,3-beta-glucanosyltransferase	PHR2
	A0A2H1A768	Multidrug resistance protein CDR1	CDR1
	A0A2H0ZGL1	Uncharacterized protein	
B8441	A0A2H1A1L1	60S ribosomal protein L16-B	RPL16A
	A0A2H0ZVG5	Asparagine synthase (glutamine-hydrolyzing)	ASN1
	A0A2H0ZC49	Aconitate hydratase, mitochondrial	ACO1
	A0A2H0ZQG0	Ribosomal protein	RPL10A
	A0A2H0ZUP3	60S ribosomal protein L27	RPL27A
	A0A2H0ZIP1	Alcohol dehydrogenase 1	ADH1
	A0A2H1A212	S-adenosylmethionine synthase	SAM2
	A0A2H0ZDF6	60S ribosomal protein L12-A	RPL12
	A0A2H0ZQV3	UTP--glucose-1-phosphate uridylyltransferase	UGP1
	A0A2H0ZNX4	Ribosomal protein L19	RPL19A
	A0A2H0ZGB6	40S ribosomal protein S25	RPS25B
	A0A2H0ZKG4	60S ribosomal protein L24	RPL24A
	A0A2H1A5C8	40S ribosomal protein S14	RPS14B
	A0A2H0ZD52	60S ribosomal protein L20-A	RPL29A
	A0A2H0ZT69	ATP synthase subunit beta	ATP2
	A0A2H0ZCW1	Mannose-1-phosphate guanyltransferase	MPG1

The immunogenic proteins are highlighted in blue. The proteins identified in the cell under the same conditions are highlighted in light yellow.

## Data Availability

The RNA-seq data have been deposited at the Sequence Read Archive (SRA) database under the accession number (SRA: SRP295539 BioProject: PRJNA682185).

## Supplementary Materials

The following supporting information can be downloaded at: https://www.mdpi.com/article/10.3390/jof8100990/s1, Figure S1: EV production upon amphotericin B treatment.; Table S1: mRNA and ncRNA data common to all strains. Table S2: mRNA and ncRNA data common to at least 2 strains. Table S3: mRNA and ncRNA derived from B8441 strain. Table S4: mRNA and ncRNA derived from B11244 strain. Table S5: mRNA and ncRNA derived from MMC1 strain. Table S6: asRNA data. Table S7: proteomics data.
