# Supplementary material for: Caspofungin Affects Extracellular Vesicle Production and Cargo in Candida auris
Source: J Fungi (Basel). 2022 Sep 21;8(10):990. doi: 10.3390/jof8100990 (PMC9605528; doi:10.3390/jof8100990)
Supplement: Supplementary file 1 [file jof-08-00990-s001.zip › Figure S1.pdf]

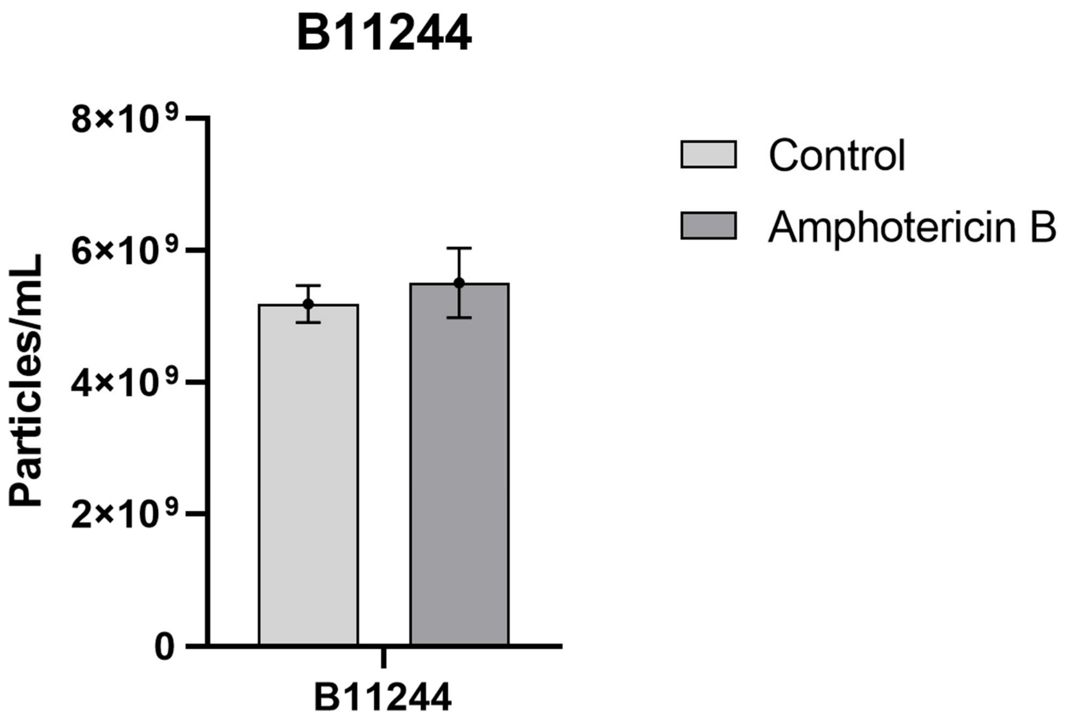

Figure S1. Representative comparison concentration distribution for B11244 EVs from control (light grey) and Amphotericin B treatment (dark grey). The average EV concentration value (y-axis) is indicated in particles/mL. The X-axis indicate the strains studied. No significance was found with t-tests followed by Bonferroni's test for three independent experiments (alpha = 0.05).
